# Interaction of genetic variants and methylation in transcript-level expression regulation in Alzheimer’s disease by multi-omics data analysis

**DOI:** 10.1186/s12864-025-11362-x

**Published:** 2025-02-20

**Authors:** Seonggyun Han, Soo-ah Cho, Wongyung Choi, Karen Eilbeck, Hilary Coon, Kwangsik Nho, Younghee Lee

**Affiliations:** 1 Department of Biomedical Informatics, University of Utah School of Medicine, Salt Lake City, UT USA; 2 Department of Psychiatry & Huntsman Mental Health Institute, University of Utah School of Medicine, Salt Lake City, UT USA; 3 The Research Institute for Veterinary Science, College of Veterinary Medicine, Seoul National University, Seoul, 08826 South Korea; 4 Center for Neuroimaging, Department of Radiology and Imaging Sciences and Indiana Alzheimer’s Disease Research Center, Indiana University School of Medicine, Indianapolis, IN USA

**Keywords:** Alzheimer’s disease, SNP, Methylation, Genetic regulation, Epigenetic regulation

## Abstract

**Background:**

Alzheimer’s disease (AD) presents a significant public health problem and major cause of dementia. Not only genetic but epigenetic factors contribute to complex and heterogeneous molecular mechanisms underlying AD risk; in particular, single nucleotide polymorphisms (SNPs) and DNA methylation can lead to dysregulation of gene expression in the AD brain. Each of these regulators has been independently studied well in AD progression, however, their interactive roles, particularly when they are located differently, still remains unclear. Here, we aimed to explore the interplay between SNPs and DNA methylation in regulating transcript expression levels in the AD brain through an integrative analysis of whole-genome sequencing, RNA-seq, and methylation data measured from the dorsolateral prefrontal cortex.

**Results:**

We identified 179 SNP-methylation combination pairs that showed statistically significant interactions associated with the expression of 67 transcripts (63 unique genes), enriched in functional pathways, including immune-related and post-synaptic assembly pathways. Particularly, a number of HLA family genes (*HLA-A*, *HLA-B*, *HLA-C*, *HLA-DRB1*, *HLA-DRB5*, *HLA-DPA1*, *HLA-K*, *HLA-DQB1*, and *HLA-DMA*) were observed as having expression changes associated with the interplay.

**Conclusions:**

Our findings especially implicate immune-related pathways as targets of these regulatory interactions. SNP-methylation interactions may thus contribute to the molecular complexity underlying immune-related pathogenies in AD patients. Our study provides a new molecular knowledge in the context of the interplay between genetic and epigenetic regulations, in that it concerns transcript expression status in AD.

**Supplementary Information:**

The online version contains supplementary material available at 10.1186/s12864-025-11362-x.

## Background

Alzheimer’s disease (AD) is one of the most complex neurodegenerative diseases and as such, is underpinned by a number of heterogeneous biological factors [1, 2]. In particular, not just genetic factors (i.e., single nucleotide polymorphisms; SNPs) but also epigenetic factors (i.e., DNA methylation) play significant roles in the neuropathogenesis of AD [3–5].

Previous twin and family studies have shown that genetic inheritance significantly contributes to AD development, accounting for an estimated 60 ~ 80% of the heritability [6], identifying 89 SNPs (*p* < 10e-5) were identified by a previous twin study [7]. In addition, genome-wide association studies (GWAS) have successfully discovered many AD-risk SNPs, giving concrete form to those implicated genetic factors [8, 9], exhibiting 8.9 ~ 31.2% of SNP-based heritability [10–13]. For example, in two different large-scale meta GWAS with 788,989 and 455,258 respective participants, including AD patients [5, 14], a recent study revealed 42 new AD-associated SNPs. That is to say, a considerable portion of individual difference in AD risk could be determined by genetic susceptibility.

Extensive epigenetic differences have also been commonly observed in the brains of human AD patients, particularly a broad constellation of gene-specific DNA methylation states that differ between brain regions, as befits the status of DNA methylation as a major epigenetic modality [4, 15, 16]. For example, the AD-risk genes *ANK1* and *BIN1* have been shown to have differential DNA methylation [17]. Most notably, the DNA methylation changes associated with AD risk are observed in early stages of the disease, which suggests that abnormal DNA methylation might contribute to disease progression. More recently, a genome-wide study of DNA in AD identified 118 differential methylation sites that are enriched in neurogenesis-related functional pathways [18]. These results suggest that altered DNA methylation is an additional important factor for the molecular mechanisms underlying AD development [18, 19].

A considerable body of evidence has shown that neurological-related functional pathways depend upon tight regulation and coordination of gene expression, and dysregulation of gene expression occurs in the onset and development of AD [20–24]. Both SNPs and DNA methylation are involved in regulating gene transcription by affecting the binding affinities of transcription factors (TFs) in regulatory regions (i.e., promoter regions) [25, 26]. Accordingly, studies have been undertaken to explore the effects of SNPs (expression quantitative trait loci; eQTL) and methylation sites (me-QTLs) on gene expression changes [27, 28]. One recent study identified 274 significant SNPs that influence 20 AD-risk genes via eQTLs [29]. Other studies have highlighted SNP-regulated expressions in AD-risk genes including *PLIN2* [24], *MS4A* [30], *MAPT* [31], and *CR1* [32]. Finally, a substantial proportion (62%) of differentially methylated regions are associated with dysregulation of gene expression in AD, having potential roles of immune process-related pathways [33].

Genetic and epigenetic factors have each independently received intensive study for their roles in gene expression regulation [34, 35]. However, it is also well-established that SNPs and DNA methylation within regulatory regions interact with each other to regulate gene expression patterns in an integrative manner [36]. For example, genetic variants may show different associations with expression levels depending on whether DNA methylation levels are high or low. Thus, in a given regulatory region, the spatial interaction between a SNP and DNA methylation could be functionally important, and investigation of these SNP-methylation (G x M) interactions could advance our understanding of the genetic and epigenetic architecture of gene expression regulation. In fact, a previous study that performed an interaction analysis for SNPs and DNA methylation discovered nine novel differentially methylated sites in genes such as VEGFA and HLA-DRB1, which sites were critically associated with congenital heart defects [37]. Another study identified SNP-DNA methylation pairs in candidate genes statistically associated with several disease phenotypes, including asthma [38, 39]. However, there is to date no study of such G x M interaction effects on gene expression in AD patients.

Here, we first seek to investigate the regulatory effect of G x M in regulatory regions (i.e., exclusively in promoter regions) on gene expression by means of integrative multi-omics analysis of whole-genome sequencing (WGS), RNA-seq, and methylation array data generated from the ROS and MAP (ROSMAP) project [40] in the Accelerating Medicines Partnership Alzheimer’s Disease (AMP-AD) program. This matched multi-omics data (*n* = 361) were measured from the dorsolateral prefrontal cortex (DLPFC) region in AD patients.

## Methods

**Fig. 1:**
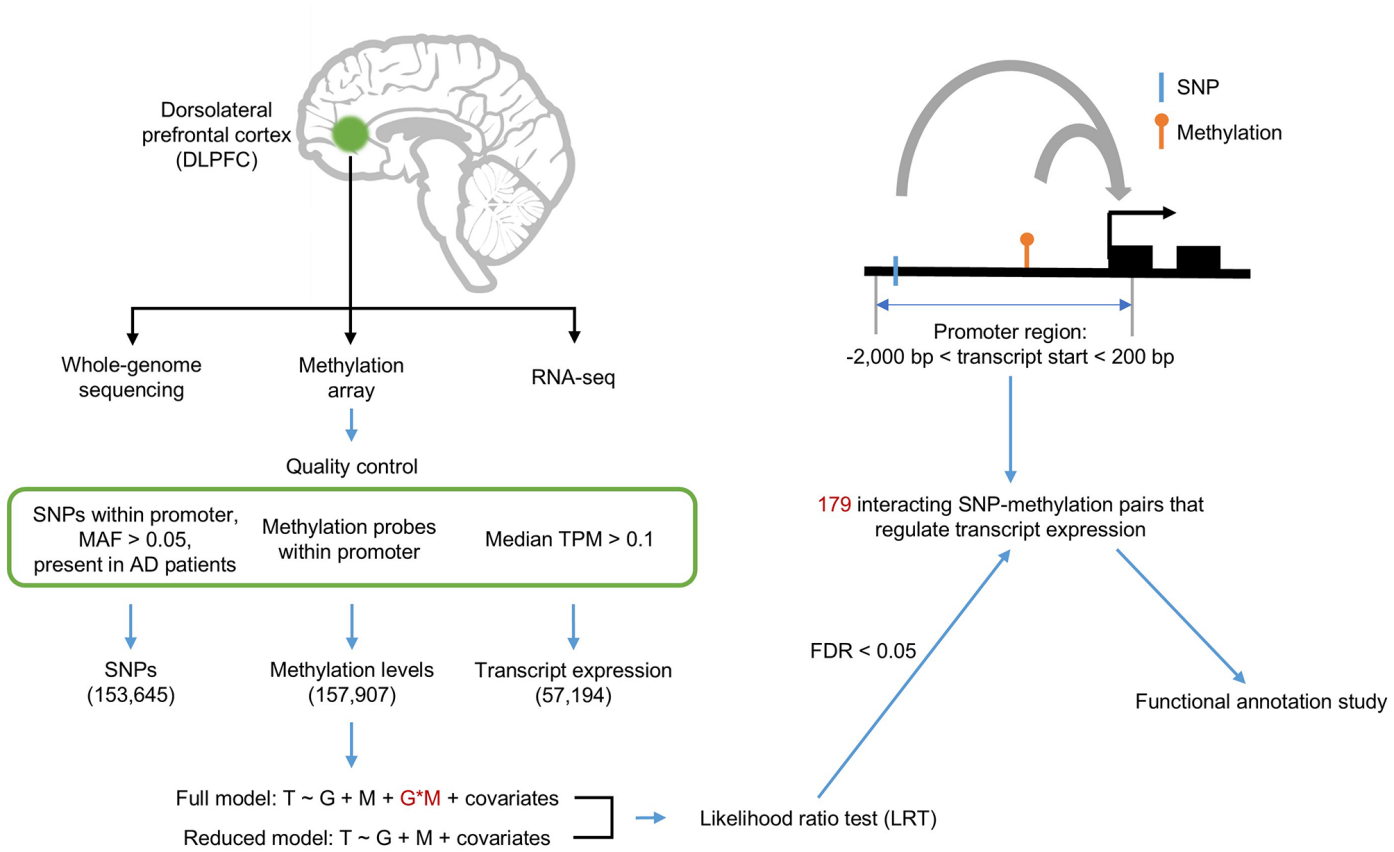
Overview of the research design. Multi-omics data including whole-genome sequencing, methylation array, and RNA-seq were generated from the dorsolateral prefrontal cortex. After quality control, linear regression with a full model and a reduced model was performed, and the results compared using the likelihood ratio test (LRT) to identify significant SNP-methylation interactions associated with transcript expression. FDR < 0.05 was the cutoff for significance. The full model is composed of G, M, and G*M interaction terms, and the reduced model of only G and M without the G*M interaction term. The promoter region was defined as spanning from − 2,000 bp upstream to 200 bp downstream from a gene’s transcription start site. AD: Alzheimer’s disease, T: transcript expression, G: genotype, M: methylation status, covariates: sex and age, MAF: minor allele frequency, FDR: false discovery rate

The overall data analysis design summary is described in Fig. 1. Integrative analysis of WGS, RNA-seq, and methylation data was performed to identify SNP-methylation pairs whose interaction had significant effect on transcript expression in AD. For example, AD samples were defined with CEREAD < = 3, which corresponds to a possible AD diagnosis in order to maximize the AD sample size. Detailed demographic information of the analyzed samples is presented in Additional file 1: Table S1.

### Omics data

We previously published a webtool, ADAS-viewer [41], to provide functions for visualizing and analyzing multi-omics data, including WGS, RNA-seq, and methylation data from the brains of human AD and control samples. We utilized those pre-processed data in this study.

### RNA-seq data

As presented in our previous publication, the RNA-seq dataset was downloaded from the Synapse database (www.synapse.org). It was generated by the ROSMAP study [40] (Synapse id: syn3388564) in the AMP-AD project. RNA-seq files were mapped by STAR aligner v2.5 [42], and transcript expression as TPM (transcripts per million) was estimated using RSEM v.1.3.0 [43]. For the aligning and estimating, we used a reference GTF file generated from Ensembl (GRCh37.75 based on hg19, http://ftp://ftp.ensembl.org/pub/release-75/gtf/homo_sapiens).

### Whole genome sequencing (WGS) data

We downloaded per-chromosome vcf files processed from WGS, including matched samples with the RNA-seq dataset presented above. These files were likewise generated from the AMP-AD project (Synapse id: syn11707419). The raw WGS data was mapped to the GRCh37 human reference using Burrows-Wheeler Aligner (BWA-MEM v0.7.8) [44]. After marking of duplicate reads by Picard tools v.1.83 (http://broadinstitute.github.io/picard/), the data were locally realigned around indels, base quality score recalibration (BQSR) performed, and variants called by the Genome Analysis Toolkit (GATK v3.4.0) [45].

### Methylation data

Methylation data including matched samples with RNA-seq and WGS were generated by the ROSMAP study using the Illumina InfiniumHumanMethylation450 bead chip assay, and we obtained this data from Synapse (Synapse id: syn3157275). The data was adjusted and normalized for age, sex, and experimental batch.

### Identification of interacting SNP and methylation pairs

We examined SNPs and methylation probes within promoter regions (+ 2,000 to -200 bps from transcription start site). Quality control processing was performed on the data. We analyzed only SNPs within promoter, minor allele frequency > 0.05, present in AD patients and transcripts with median TPM > 0.1 across all samples. To identify significant interactions, we compared the reduced and full models below by ANOVA with the likelihood ratio test (LRT).

Reduced model: T ~ G + M + sex + age.

Full model: T ~ G + M + G*M + sex + age.

In the equations, T is transcript expression, while G and M are genotype and methylation level, respectively. G*M is the interaction term in the full model. Sex and age are included as covariates. Although methylation data was adjusted by sex and age, we included these in the model to minimize any potential confounding effects since there remains the possibility that age and sex could act as confounders in the associations between expression and SNPs. We identified interaction pairs as significant based on an adjusted *p*-value, the false discovery rate (FDR), with a value < 0.05. After obtaining significant interaction pairs, we performed linkage disequilibrium (LD)-based clumping based on the CEU reference panel, retaining the interaction pair with the lowest *p*-value within each LD block of r^2^ ≥ 0.8.

### Enrichment of interaction pairs in regulatory regions

Interacting SNP and methylation pairs that have a combined role in gene expression regulation could coincide with regulatory elements such as DNase hypersensitive sites. We evaluated whether our SNPs were enriched in regulatory regions by utilizing RegulomeDB [46], a database that includes SNP functionality scores (range 1 to 6) based on available evidence of regulatory potential. A lower score indicates that SNPs are more likely to affect transcript factor binding in regulatory regions. We further explored the enrichment of our SNPs and methylation sites in specific DNA accessibility and histone modification occupancy footprints that indicate critical regulatory regions. A comprehensive set of these regulatory regions was obtained by collecting different ChiP-seq data (i.e., H3K4me3, H3K27me3, H3K27ac, and DNase-seq) measured from the middle frontal area of the AD brain in the Encyclopedia of DNA Elements project [47]. We downloaded processed result data (i.e., a bed file of narrow peaks including open chromatin regions) and determined the enrichment of SNP and methylation interaction pairs in each region set using Fisher’s exact test.

### Identification of the functional implications of genes regulated by interactions

Functional over-representation analysis to evaluate the functional roles of genes regulated by SNP-DNA methylation interplay was carried out using ConcensusPathDB (CPDB) [48] with databases including the Reactome database and Gene Ontology (GO) terms. We defined significantly enriched pathways and GO terms as those having FDR q-values < 0.05. We further constructed a protein-protein interaction (PPI) network for all regulated gene sets using the StringDB web tool [49].

### Differential gene expression between AD patients and cognitively normal individuals

We compared gene expression between AD patients (*n* = 361) and cognitively normal (CN) individuals (*n* = 133) by performing logistic binomial regression, with adjustment for sex and age.

### Replication study with omics data from an independent cohort

To replicate our significant interaction pairs, we utilized independent cohort omics data measured from the human parahippocampal gyrus (PHG) in AD patients (*n* = 121). The dataset was generated from the MSBB project [50] in AMP-AD, a joint study with the one that generated our primary analysis data (ROSMAP). These samples are described in Additional file 1: Table S1, and all methods relating to processing and statistics were consistent with those of our primary analysis. FDR < 0.1 was used to define replication significance.

## Results

### Identification of SNP-DNA methylation interaction pairs in AD

**Fig. 2:**
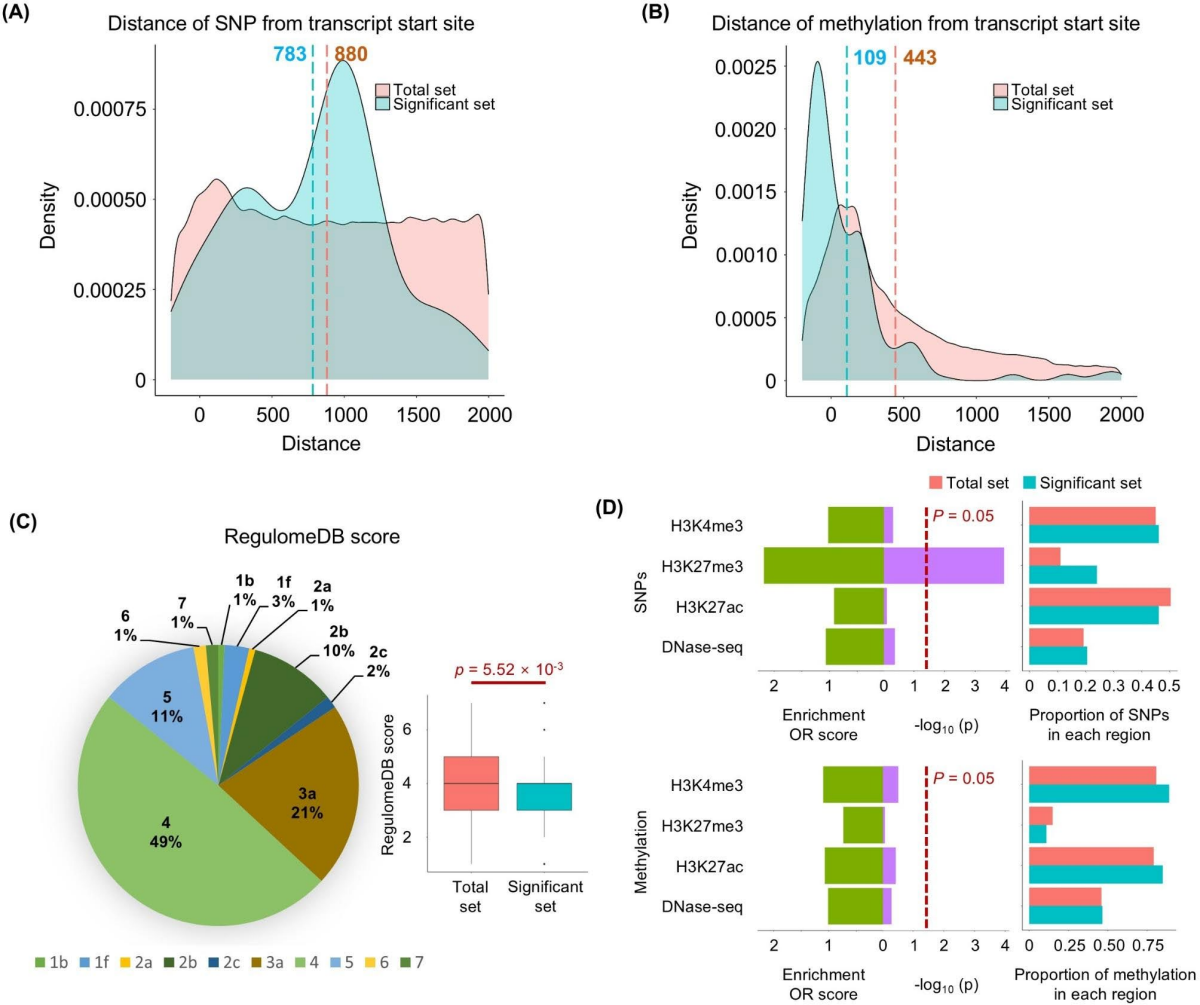
Context profiling of SNP and methylation interaction pairs. Distance of (**A**) SNP and (**B**) methylation probes from transcript start site among the total and significant sets. (**C**) Pie chart of the RegulomeDB score distribution of all SNPs, and boxplot summarizing respective scores among the total and significant sets; *p*-value estimated by Wilcoxon rank-sum test. (**D**) Left bar plots show the enrichment odds ratio and -log_10_*p*-value obtained by comparing the number of SNPs (top) or methylation sites (bottom) located within peak regions corresponding to the given regulatory region marker between the total and significant sets; *p*-values were calculated with a 2 × 2 Fisher’s exact test. The right bar plot indicates the proportion of all SNPs or methylation sites within each regulatory region type

After the pre-processing for quality control, we retained and tested 153,645 SNPs, 157,907 methylation probes, and 57,194 transcripts, using the LRT to compare full and reduced models and identify pairs with significant contribution of the interaction term. We identified a total of 313 SNP-DNA methylation interaction pairs that regulate 67 transcripts (63 unique genes) having a significant difference between the models at FDR < 0.05. After LD clumping, 179 SNP-methylation interaction pairs remained, comprising 141 unique SNPs and 73 methylation probes (Additional file 1: Table S2). We first profiled the distances of paired SNPs and methylation sites from transcription start sites (TSS). As shown in Fig. 2A, there was no significant difference in distance from TSS between SNPs in the total set and those in significant pairs, while methylation probes of the significant pairs tended to be within promoter regions and closer to the TSS (Fig. 2B); the average distance from the TSS was 109 base pairs (bp) and 443 bp for methylation sites in the significant and total sets, respectively. Moreover, compared to the total set, the interaction pairs were enriched for methylation probes located closer to the transcript TSS than their corresponding SNP (Additional file 2: Figure S1).

To identify SNPs with regulatory functionality, we evaluated whether our SNPs were enriched in regulatory regions using RegulomeDB, a database that includes SNP functionality scores (scale 1 to 6) based on evidence for their regulatory potential. Approximately 38% of significant SNPs had RegulomeDB scores of 3 or less, which is suggestive of regulatory function; this proportion was elevated compared to the total set of tested SNPs (31%, *p* = 5.52 × 10^− 3^ e-3 by Wilcoxon rank-sum test) (Fig. 2C). Further analysis against histone modification and DNase ChiP-seq data revealed the SNPs of interaction pairs to be significantly enriched among H3K27me3 peak regions (*p* = 1.16 × 10^− 4^ and odds ratio = 2.18) (Fig. 2D). These results suggest that our significant SNP-methylation pairs tend to occur in regulatory elements that affect gene expression.

### SNP-DNA methylation crosstalk regulates immune-related genes

**Fig. 3:**
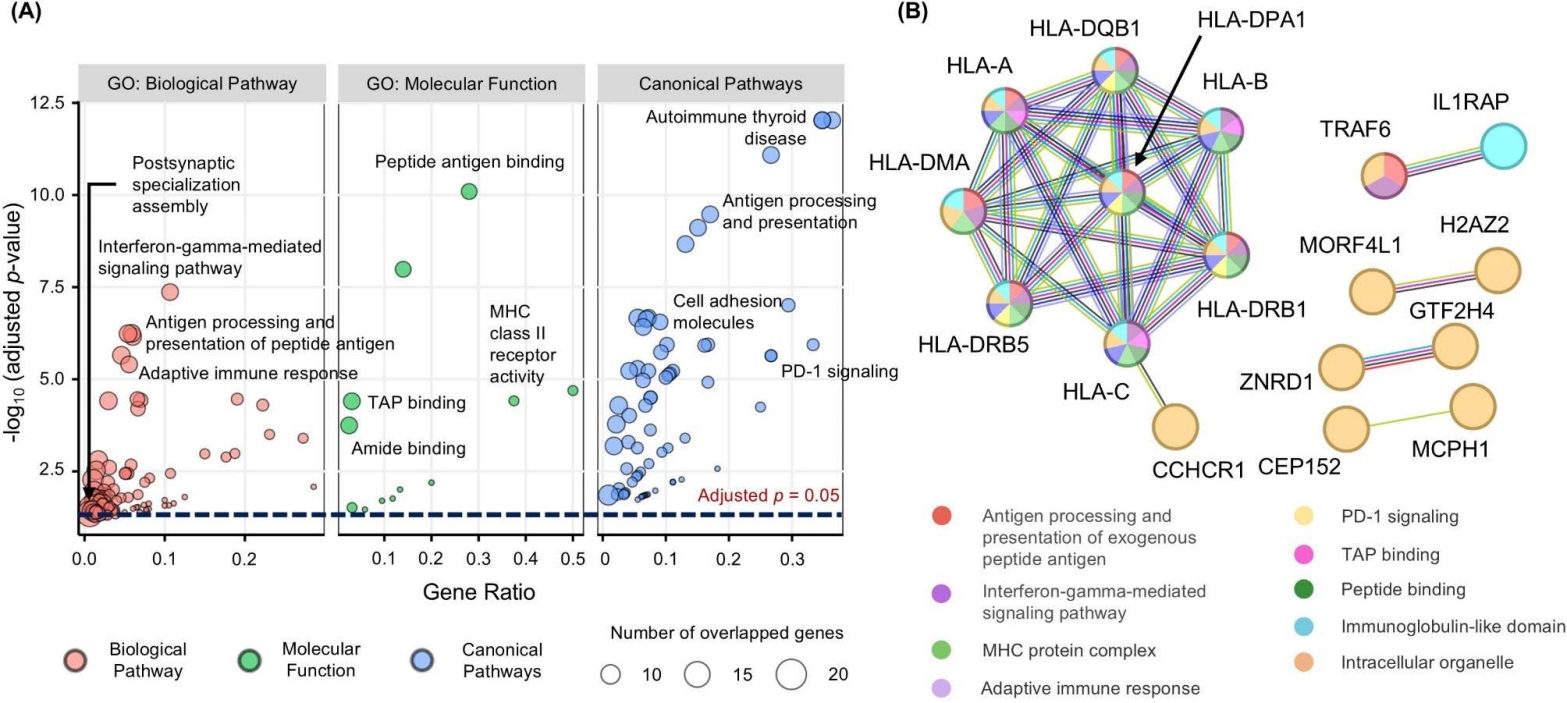
Enriched gene sets and protein-protein interaction network of genes regulated by SNP-DNA methylation interactions. (**A**) Gene Ontology terms and functional pathways enriched among gene sets associated with SNP-DNA methylation interactions. X-axis and Y-axis indicate gene ratio (fraction of term or pathway genes also in our gene set) and -log_10_ adjusted *p*-value, respectively. (**B**) Protein-protein interaction networks in our gene set, according to StringDB

To gain insight into the biological and molecular functions of the 63 genes differentially regulated by SNP-methylation pairs in AD, we carried out gene set enrichment analysis against functional pathways and the Gene Ontology (GO) database using CPDB (see Methods). We observed significant enrichment of immune-related functional pathways and GO terms, including PD-1 signaling, MHC class 1 and 2 antigen processes, interferon 1 response, and interferon gamma-related pathway (Fig. 3A and Additional file 1: Tables S1 and S4). In addition, we observed enrichment of postsynaptic assembly-related GO terms, which represent important functions for transmitting neuronal signals at central synapses; this suggests that important genes underlying AD are regulated by interactions of SNPs and DNA methylation.

We next explored the protein-protein interaction (PPI) network of the identified genes to further investigate their molecular signature (Fig. 3B). In this network, HLA family genes clustered together, with *HLA-DPA1* in the position of potentially being a hub gene that has interacted connections with many other genes.

### The majority of HLA family genes are regulated by SNP-methylation crosstalk

We found nine genes in the HLA family (human leukocyte antigen) to be controlled by SNP-DNA methylation crosstalk: *HLA-A*, *HLA-B*, *HLA-C*, *HLA-DRB1*, *HLA-DRB5*, *HLA-DPA1*, *HLA-K*, *HLA-DQB1*, and *HLA-DMA*. The total number of HLA genes in our analysis was 22, and this family represented 0.135% and 14.52% of our significant gene set and of the total set, respectively. In other words, HLA family members are very significantly enriched in our set (*p* < 1 × 10^− 6^ from chi-square test, based on a simulation analysis with 1,000,000 trials). These genes are part of the major histocompatibility complex (MHC), which is responsible for appropriately presenting antigens and so plays a significant role in human inflammatory and immune functions. Notably, we determined transcript expression of MHC class I genes including *HLA-A*, *-B*, *-C*, and *-K* to be regulated by SNP-methylation pairs via synergistic effects, while those belonging to MHC class II (*HLA-DRB1*, *HLA-DRB5*, *HLA-DPA1*, *HLA-DQB1*, and *HLA-DMA*) are controlled via anti-synergistic effects (Additional file 2: Figure S2).

### HLA-DPA1 expression is regulated by SNP-DNA methylation interaction

Since *HLA-DPA1* appears as a hub gene in our PPI network, we chose this gene as a case study with which to demonstrate how a SNP and methylation site interact to regulate transcript expression.

**Fig. 4:**
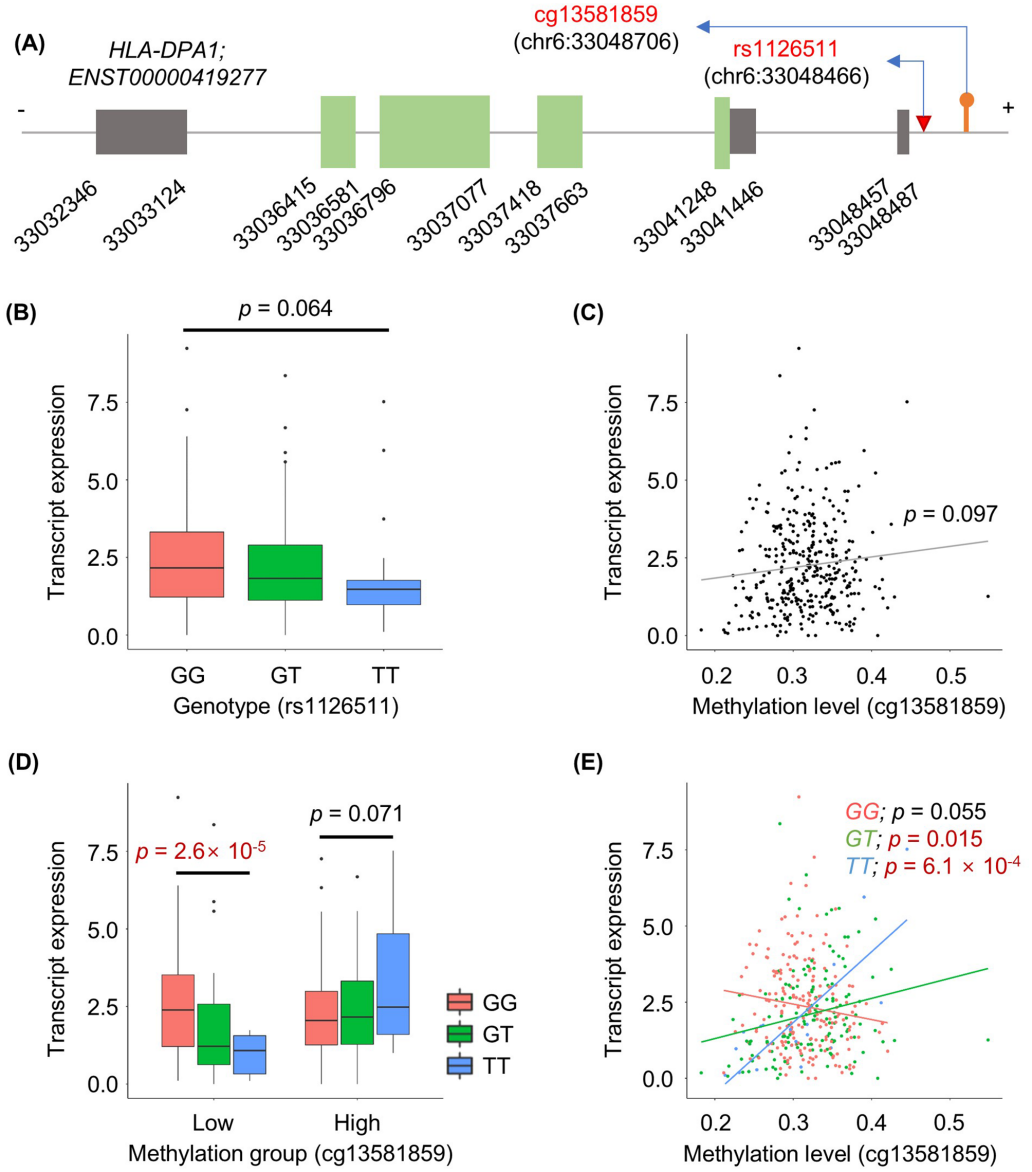
Interaction between SNP and DNA methylation status in regulating transcript expression of *HLA-DPA1*. (**A**) Gene model of ENST00000419277. Grey and green boxes indicate untranslated regions and exons, respectively. Red and orange indicators represent the SNP rs1126511 and methylation probe cg13581859, respectively. (**B**) Boxplot of transcript expression distribution according to the genotype of rs1126511. (**C**) Scatter plot of transcript expression association with methylation level at cg13581859. (**D**) Boxplot of transcript expression distribution according to genotype among low- and high-methylation groups. K-means clustering was used to classify groups. (**E**) Scatter plot of transcript expression association with methylation level for each genotype

The SNP and methylation sites in question are rs1126511; chr6:33048466 and cg13581859; chr6:33048706, located in the promoter of transcript ENST00000419277 of *HLA-DPA1* (Fig. 4A). By themselves, neither the SNP genotype nor methylation status are significantly associated with transcript expression (Fig. 4B and C), whereas their combination shows significant association with expression (Fig. 4D and E). Under low methylation status, the *T* allele tends to decrease expression, but not under high methylation (Fig. 4D); that is, the SNP effect is modulated by methylation status. Additionally, high methylation promotes increased expression in conjunction with the *T* allele, but not with the *G* allele. Therefore, *HLA-DPA1* transcript expression in AD patients is most decreased under the combination of the *T* allele and low methylation.

As *HLA-DPA1* is upstream of a cell-surface signaling pathway, we further explored the downstream impact of this regulation of *HLA-DPA1* transcript expression. Specifically, we performed differential gene expression analysis to compare between AD patients with high (greater than the third quartile) and low (less than the first quartile) expression of *HLA-DPA1*. We identified 1,285 significantly differentially expressed transcripts with Bonferroni-corrected *p* < 0.05 and fold change > 2 (Additional file 1: Table S5). Among these genes, over-representation analysis identified enrichment of immune-related functional pathways, including immune response, inflammatory response, and cytokine binding (Additional file 2: Figure S3).

### Differentially expressed genes between AD and cognitively normal individuals

**Fig. 5:**
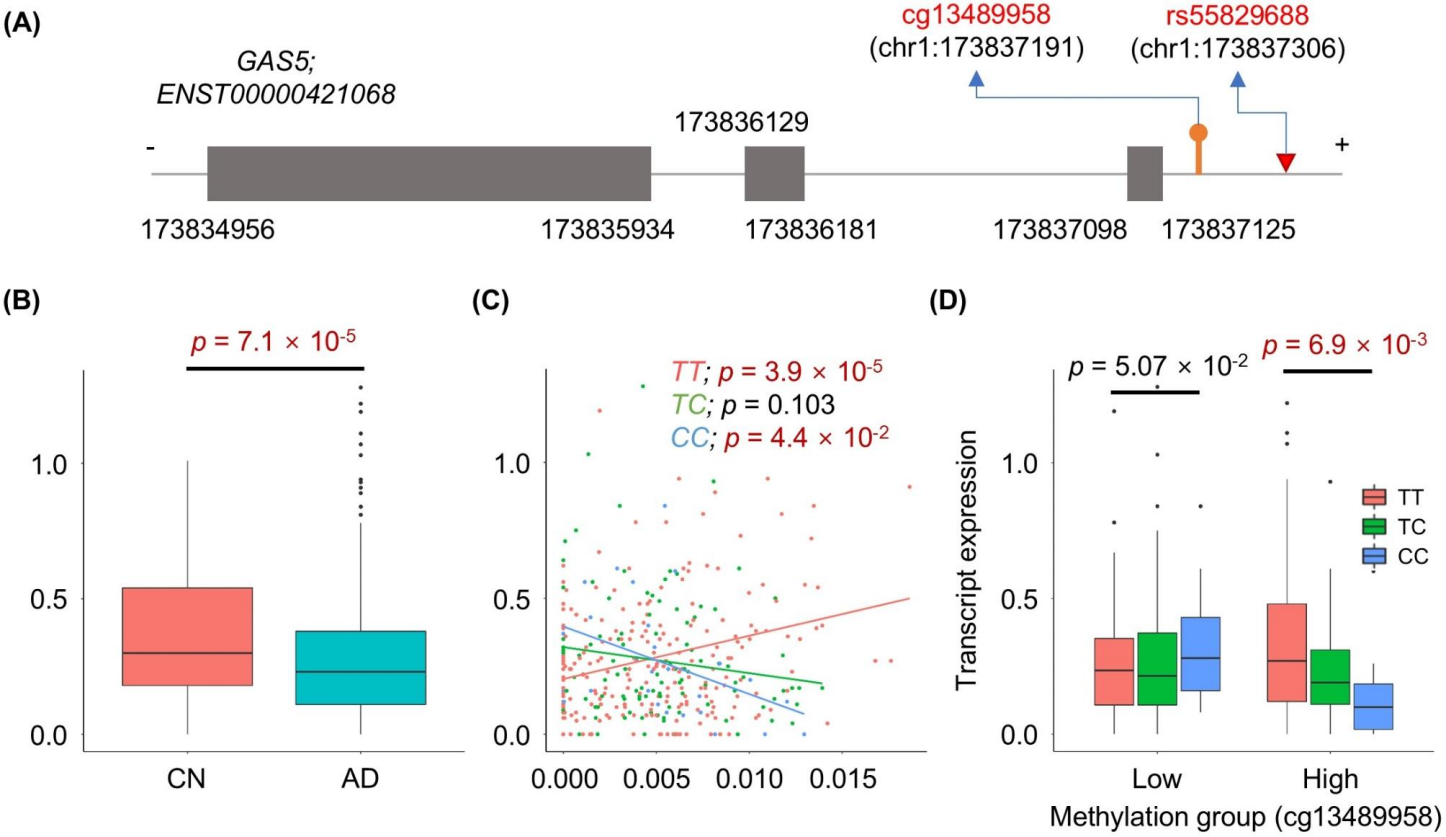
Differentially expressed *GAS5* between cognitively normal (CN) and AD, regulated by SNP-DNA methylation interaction. (**A**) Gene model of ENST00000421068; it is RNA gene: grey boxes indicate untranslated regions of exons. Red and orange indicators represent the SNP rs55829688 SNP and methylation probe cg13489958, respectively. (**B**) Boxplot of transcript expression distribution according to disease status. (**C**) Scatter plot of transcript expression association with methylation level for each genotype. (**D**) Boxplot of transcript expression distribution according to genotype among low- and high-methylation groups. K-means clustering was used to classify groups

Of the interaction-regulated genes, DEG analysis identified 14 genes as differentially expressed between AD and CN individuals (Additional file 1: Table S6). Notably, *GAS5* (growth arrest specific 5) transcript ENST00000421068 (Fig. 5A) was the most significantly down-regulated in the DLPFC brain region of AD patients compared to CN individuals (Fig. 5B). This expression change was associated with an interaction of the SNP rs55829688 (chr1:173837306) and methylation status at cg13489958 (chr1:173837191) (Fig. 5C and D). Under high methylation, expression of *GAS5* tends to be decreased by the *C* allele of the SNP, while under low methylation it is increased. In addition, expression level in the high-methylation group varies with SNP genotype, increasing and decreasing with *TT* and *CC*, respectively. Transcript expression was not significantly associated with rs55829688 genotype alone (Additional file 2: Figure S4). Thus, AD patients with both the *CC* genotype at rs55829688 and high methylation level at cg13489958 have low expression of *GAS5* in the DLPFC.

### Replication study in an independent cohort dataset

Analysis of an independent cohort dataset (different brain region, see the Methods) replicated 75 interaction pairs regulating expression of ten transcripts (Additional file 1: Table S7), approximately 42% of the pairs (15% of transcripts) identified by our primary analysis. Moreover, this analysis replicated three HLA family genes (*HLA-K*, *HLA-DRB1*, and *HLA-DQB1*) as being regulated by SNP-methylation interactions.

### Association models considering other covariates

Since methylation data was corrected for age and sex, it is possibility that our primary ANOVA model including age and sex as covariates may potentially lead to over-correction. Therefore, we re-ran the ANOVA model excluding age and sex as covariates, specifically for the set of significant interaction pairs. All pairs remained significant, and the correlation coefficient between the -log_10_(p-values) from the model with and without age/sex was 0.997 (Additional file 1: Table S8 and Additional file 2: Figure S5). In addition, as the clinical features, such as Braak stage, CEREAD, and APOE4 status, are important factors in AD, we also re-ran the ANOVA model, including Braak stage, CEREAD, and APOE4 status as covariates. All pairs remained significant, and the correlation coefficient between the -log_10_(p-values) from the model with and without these factors was 0.998 (Additional file 1: Table S9 and Additional file 2: Figure S6).

## Discussion

In this study, we explored the crosstalk between non-co-located SNPs and DNA methylation in regulating transcript-level expression in AD. To our knowledge, this is the first study designed to investigate such interactions at the genome-wide level in AD patients. Since transcriptional regulation in neurodegenerative diseases is both genetically and epigenetically complex [8, 33, 51, 52], our strategy of discovering SNP-DNA methylation interactions that affect gene expression is essential for advancing our understanding of the molecular architecture underlying AD, aspects of which could be missed by focusing on either genetic or epigenetic factors alone, as has been discovered in previous integrative analyses [36–39]. A prior study successfully determined the influence of SNP rs3024685 on asthma risk to be modulated by DNA methylation at a separate site, cg09791102: the effect of the SNP on risk was increased in individuals with higher methylation [39]. In addition, another more recent genome-wide study found DNA methylation levels to modify the SNP-associated genetic risk of orofacial clefts, a congenital malformation [36]. As demonstrated by these studies of other conditions, investigating the joint contributions of genetic and epigenetic mechanisms to gene expression regulation could be useful for providing new insights into the underlying mechanisms of AD.

Our analysis revealed that, after LD clumping, 179 SNP-DNA methylation pairs were significantly associated with the expression of 63 genes in AD patients. Functional enrichment analysis supported that these genes are closely related with AD neurological progression, with enrichments observed for: immune-related pathways including innate immune response, the adaptive immune system, interferon alpha/beta and gamma signaling, PD-1 signaling, and regulation of interleukin-6 production. AD has hallmark neuropathological features in the accumulation of amyloid beta (Ab) plaques and neurofibrillary tangles, and also in neuroinflammation [20, 53]. The immune system is a key player in restricting Ab and tau toxicity through clearing debris and toxic materials from the brain, and the innate immune pathway in microglia cells (i.e., immune cells that contribute to initiation of neuroimmune responses) is known to especially contribute to Ab phagocytosis, promoting its clearance and proper neurogenesis [54–58]. Proteins in this pathway act as a barrier that envelops Ab plaques and prevents further aggregation. Additionally, the complement proteins (i.e., C1, C3, and C5), which are also part of the innate immune system, contribute to AD progression through their involvement in synaptic pruning and Ab phagocytosis, as well as connecting with neurons and astrocytes [59, 60]. For example, complement protein inhibitors have been observed to cause significantly higher Ab peptide deposition and lessen the phagocytic phenotype, leading to neuron degeneration and synapse loss [61, 62]. A third important factor in AD pathology is adaptive immune function, with the interferon and interleukin signaling pathways being associated with microglia cell activity and synapse loss [55, 57, 58, 63]. Finally, PD-1 also a critical factor in microglia, as it contributes to increasing Ab plaque deposition and neuroinflammation and interacts with PD-L1 in astrocytes to be induced by the AD-risk genes *ADAM17* and *BACE1* [64].

Interestingly, nine of *HLA* family genes were observed in our interaction-regulated set; this represents a remarkably high enrichment over background, which suggests that regulatory interactions may be especially important for HLA-related pathways. Genes in the *HLA* complex have reported associations with susceptibility to neurodegenerative diseases including AD and Parkinson’s disease (PD) [65–67]. Being involved in essential aspects of human innate and adaptive immune mechanisms, these genes contribute to a wide range of neuroinflammatory activities in the brain, and hence affect brain development and plasticity in relation to AD pathogenesis [65, 68–70]. Moreover, HLA genes are linked to the process of Ab accumulation in the brain [67, 71, 72]. Microglia cells swallow Ab peptides and process them into pieces, then present the pieces in combination with HLA molecules as antigens, leading to immune reactions that are important for Ab clearance [65]. In fact, specific alleles of HLA-DR genes, which are among our significant results, affect the response of Ab-reactive T-cells in mice, and hence are important in Ab peptide autoimmunity and the clearance of amyloid plaques [73, 74]. A fine-mapping study of HLA genes as AD risk factors further demonstrated these genes, including *HLA-A* and *HLA-B* to have effects on AD risk and to specifically be associated with higher CSF (Cerebrospinal fluid) amyloid levels through their effect on neuroinflammation [75]. However, while genetic variants in the HLA complex have been spotlighted as AD risk factors linking to immune inflammatory responses, some of studies have shown discrepancies of risk associations for HLA genes [76]. For example, genetic variants of *HLA-A* have been reported as being variously associated with increased AD risk or not related to risk [75, 76]. Epigenetic modulation of the effects of SNPs in HLA genes, such as by methylation, might contribute to these inconsistent reports. Therefore, methylation status may need to be considered as a confounder when exploring the contributions of HLA variants to AD progression.

We presented as a case study the interplay of a SNP and DNA methylation pair in regulation of *HLA-DPA1* expression. In our PPI network, this gene stood out as a potential hub gene. We observed its expression in the AD brain to be lower in the joint presence of rs1126511 allele *T* and low methylation at cg13581859. Altered expression of *HLA-DPA1* has been reported to affect phagocytosis and immune pathways in neurons [72, 77]. Inappropriate phagocytosis in microglia due to lower expression of *HLA-DPA1* could result in dysregulation of debris clearance and degradation in neurons, including of Ab and tau proteins, leading to neuropathologic progression [72, 78]. Analysis of downstream target genes associated with *HLA-DPA1* expression found such genes to be significantly enriched in immune-related and inflammation pathways (Additional file 2: Figure S3), supporting that reduced *HLA-DPA1* expression due to this SNP-methylation interaction could influence immune function in AD patients. However, we did not find a statistically significant difference in *HLA-DPA1* expression according to disease status (AD vs. CN, data not shown). Therefore, further study validating this interactive regulation of an HLA gene and functionally linking it to the neurological and clinical features of AD is required.

DEG analysis found 14 interaction-regulated genes, such as *GAS5*, that were differentially expressed between AD and CN groups. *GAS5* produces growth-arrest specific transcript 5, a long noncoding RNA (IncRNA) that could play an integral part in the manifestation of human diseases [79]. A previous study revealed *GAS5* levels to be consistently reduced in human AD brain samples, and furthermore that depletion of *GAS5* decreases insulin signaling in neurons and increases phosphorylated tau, which influences synaptic dysfunction in tauopathies [79]. Our findings indicate that *GAS5* expression could be regulated by a combination of the SNP rs55829688 and the methylation site cg13489958 (Fig. 5 and Additional file 2: Figure S4). Our analysis found differential expressions of other genes associated with combined SNP and methylation status. These other genes, including *HOPX* [80–82], *REPS1* [83, 84], *OSBPL3* [85], *FXYD6* [86], *CBLN3* [87], and *PDIA2* [88], have been reported to be differentially expressed in AD and to affect neurological functions. For example, *HOPX* (HOP homeobox) contributes significantly to the neurogenic process in the brain, and its expression was found to be reduced in a transgenic mouse model of AD [80–82]. *REPS1* is reported to be important for neuron morphogenesis, exerting a negative effect on cell growth. It has also been identified as a hub gene in an analysis of DEGs in AD, suggesting it to be a potential biomarker for AD [83, 84]. *MCPH1* [89] and *ATP5MC2* [90] have been identified as AD risk genes, and *BLOC1S2* is crucial for neuronal development; knock-out mice exhibit defects in cortical development and neuronal differentiation of neural progenitor cells [91]. Lastly, *TRAF6* plays a significant role in the central nervous system, and is related to AD through its induction of Ab-induced neurotoxicity and cognitive decline [92, 93]. Our results suggest these regulatory features should be investigated together in future work to precisely understand expression variation, and for potential clinical utility as a biomarker for AD.

Although we performed LD clumping to reduce the complexity of SNP relations in the interaction analysis, several genes still included many SNPs. Of particular note are *HLA-DRB5* and *HLA-DQB1*, with 21 and 45 SNPs respectively. HLA family genes are among those with the most complex LD relations; further fine-mapping analysis is required within these and other genes having high SNP counts.

Several genes were notable for having associations not only with SNP-methylation interactions, but also with SNP or methylation status alone; however, these instances could also be more effectively understood when the combination of SNP and methylation was explored. For example, although transcript expression of *IL1RAP* was associated with genotype at rs73058141 (Additional file 2: Figure S6A), the regulatory effect was also impacted by methylation: that is, the SNP genotype is significantly associated with increased expression under high methylation, but not low methylation (Additional file 2: Figure S6).

This study has several general limitations, which merit note here to avoid potential over-interpretation. First, linking of expression variation to comprehensive clinical features of AD is limited. The impact of identified genes associated with SNP-methylation interactions on immune-related pathways has been critically discussed in our study, but we found several genes that have significant different expressions between the AD and CN groups. Therefore, further study is necessary to investigate whether joint SNP-methylation regulation of these genes links to other neuropathologic features such as brain volume, neuritic plaque density, or dementia stage. Second, although we attempted to replicate our result in an independent cohort, the replication data was generated from a different brain region and the sample size was relatively small (*n* = 121). Further replication should be explored in a dataset derived from the same brain region. Finally, it remains necessary to conduct fine-mapping studies of genes that are especially complex in their genetic variation.

## Conclusions

In conclusion, our integrative multi-omics study presents a novel mechanism of joint genetic and epigenetic regulation of gene expression in AD patients. These genes, especially HLA family genes, could be dysregulated by the combination of altered DNA methylation and SNP susceptibility allele, which may ultimately affect the disease’s neurological progression. Our findings may provide a useful resource for further studies to discover genetic mechanisms in AD and other neurodegenerative diseases and may ultimately promote precision medicine by better explaining the functions of genetic and epigenetic markers.

## Electronic supplementary material

Below is the link to the electronic supplementary material.

Supplementary Material 1

Supplementary Material 2

## Data Availability

The codes for data processing and analysis are available in GitHub: https://github.com/snubmi/SNPMet-Promoter/.

## Data availability

RNA-seq, methylation, and WGS datasets that support the findings of this study were downloaded from the Accelerating Medicines Partnership for Alzheimer’s Disease (AMP-AD) Consortium that originally generated these data (https://adknowledgeportal.synapse.org/Explore/Programs/DetailsPage?Program=AMP-AD).

## Abbreviations

AD: Alzheimer’s disease
SNP: Single nucleotide polymorphism
WGS: Whole-genome sequencing
LRT: Likelihood ratio test
GWAS: Genome-wide association studies
TF: Transcription factor
eQTL: Expression quantitative trait loci
AMP-AD: Accelerating Medicines Partnership Alzheimer’s Disease
DLPFC: Dorsolateral prefrontal cortex
TPM: Transcripts per million
BQSR: Base quality score recalibration
GATK: Genome Analysis Toolkit
FDR: False discovery rate
LD: Linkage disequilibrium
CPDB: ConcensusPathDB
GO: Gene Ontology
PPI: Protein-protein interaction
CN: Cognitively normal
PHG: Parahippocampal gyrus
TSS: Transcription start sites
HLA: Human leukocyte antigen
MHC: Major histocompatibility complex
DEG: Differentially expressed gene
GAS5: Growth arrest specific 5
Ab: Amyloid beta
PD: Parkinson’s disease
CSF: Cerebrospinal fluid
lncRNA: Long noncoding RNA
SNP-methylation: G x Me
