# Supplementary material for: Interaction of genetic variants and methylation in transcript-level expression regulation in Alzheimer’s disease by multi-omics data analysis
Source: BMC Genomics. 2025 Feb 20;26:170. doi: 10.1186/s12864-025-11362-x (PMC11844006; doi:10.1186/s12864-025-11362-x)
Supplement: Supplementary file 2 — Supplementary Material 2 [file 12864_2025_11362_MOESM2_ESM.pdf]

Interaction of genetic variants and methylation in transcript-level expression regulation in Alzheimer's disease by multi-omics data analysis

Seonggyun Han [1, 2], Soo-ah Cho [3], Wongyung Choi [3], Karen Eilbeck [1], Hilary Coon [2], Kwangsik Nho [4], Younghee Lee [3, *]

[1]*Department of Biomedical Informatics, University of Utah School of Medicine, Salt Lake City, UT, USA*

[2]*Department of Psychiatry & Huntsman Mental Health Institute, University of Utah School of Medicine, Salt Lake City, UT, USA*

[3]*The Research Institute for Veterinary Science, College of Veterinary Medicine, Seoul National University, Seoul, 08826, South Korea*

[4]*Center for Neuroimaging, Department of Radiology and Imaging Sciences and Indiana Alzheimer Disease Center, Indiana University School of Medicine, Indianapolis, IN, USA*

Supplementary Figures

- **Figure S1.** Profiling of distance from transcript start site (TSS) among SNP and methylation probes of interacting pairs.
- **Figure S2.** Direction of SNP and methylation interactions (the top signals within genes) in regulating expression of HLA family transcripts.
- **Figure S3.** Genes whose expression is associated with *HLA-DPA1* transcript expression.
- **Figure S4.** Association of *GAS5* transcript ENST00000421068 expression with either of (A) genotype at the SNP rs55829688 or (B) methylation status at the probe cg13489958.
- **Figure S5.** Interaction of the SNP rs73058141 and DNA methylation at cg24004745 on *IL1RAP* transcript expression (ENST00000072516).
- **Figure S6.** A scatter plot between the -log10(p-values) from the model with (x-axis) and without (y-axis) age/sex as covariates
- **Figure S7.** A scatter plot between the -log10(p-values) from the model with and without Braak stage, CEREAD, and APOE4 status as covariates

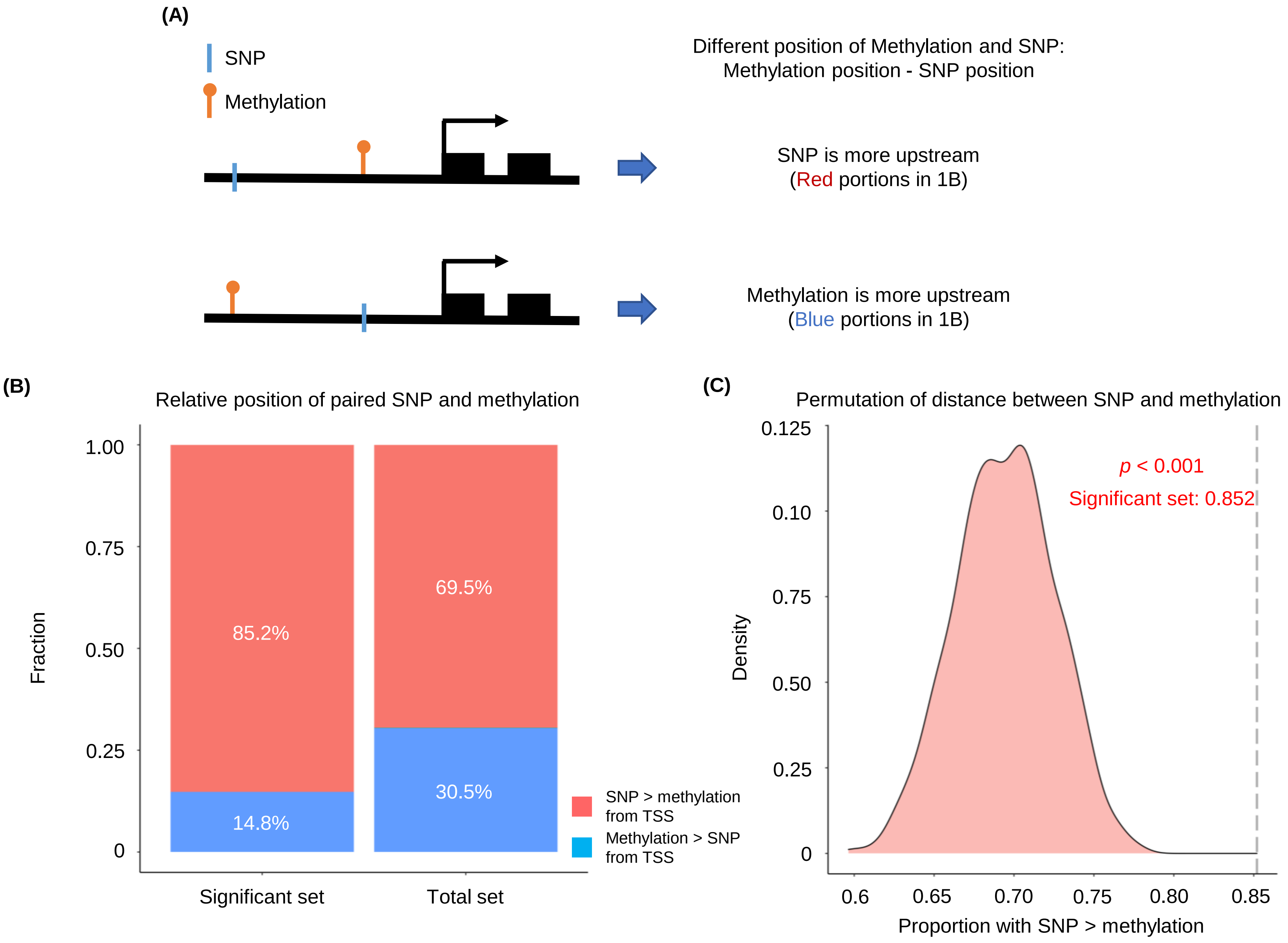

Figure S1. Profiling of distance from transcript start site (TSS) among SNP and methylation probes of interacting pairs. (A) Schematics showing relative positions of methylation probes and SNPs: methylation closer (top) and methylation further (bottom). (B) Proportion of each relative distance case from (A) in the significant set and the total set. (C) Permutation randomization of relative distances among the significant set and total set.

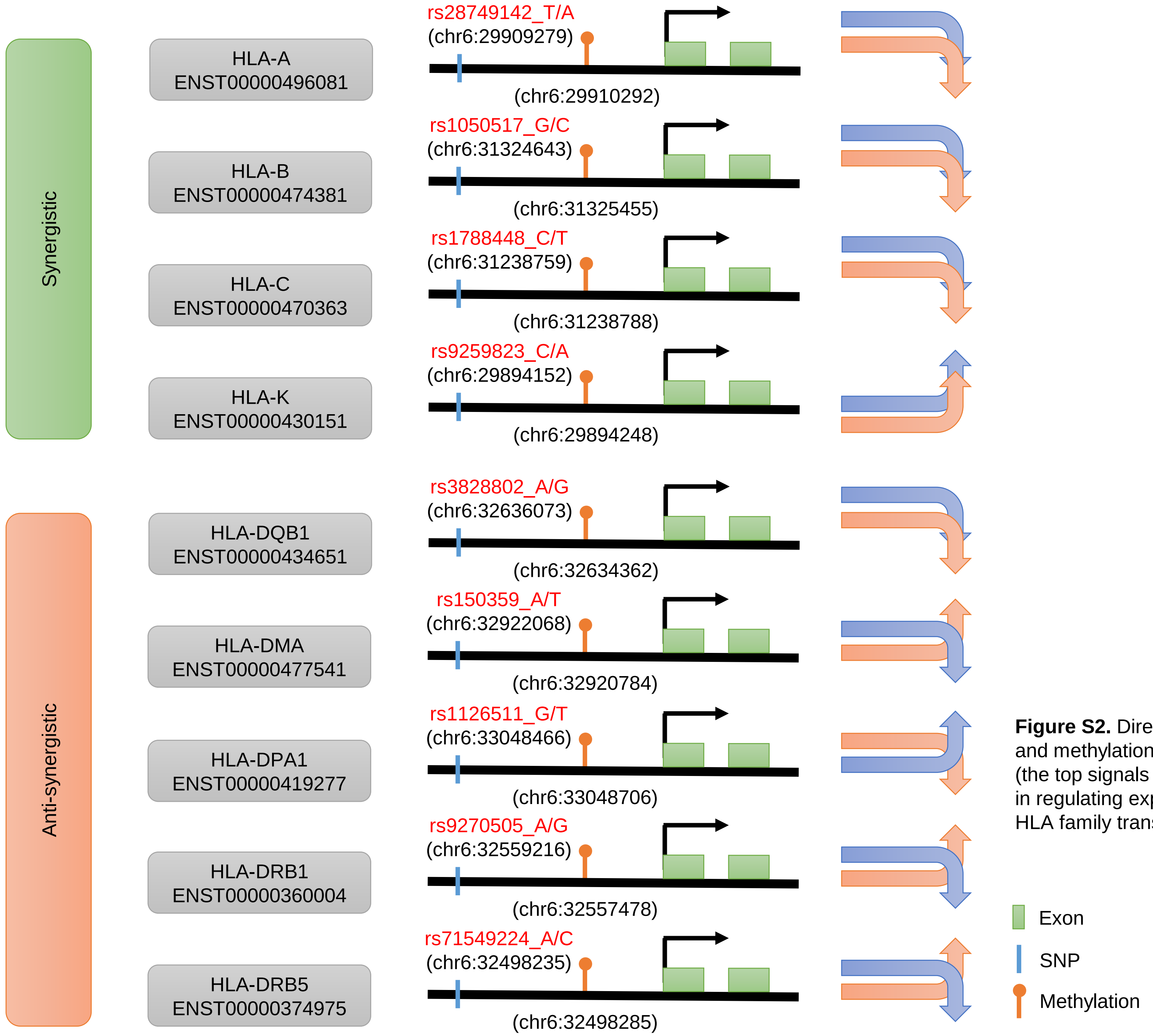

Figure S2. Direction of SNP and methylation interactions (the top signals within genes) in regulating expression of HLA family transcripts.

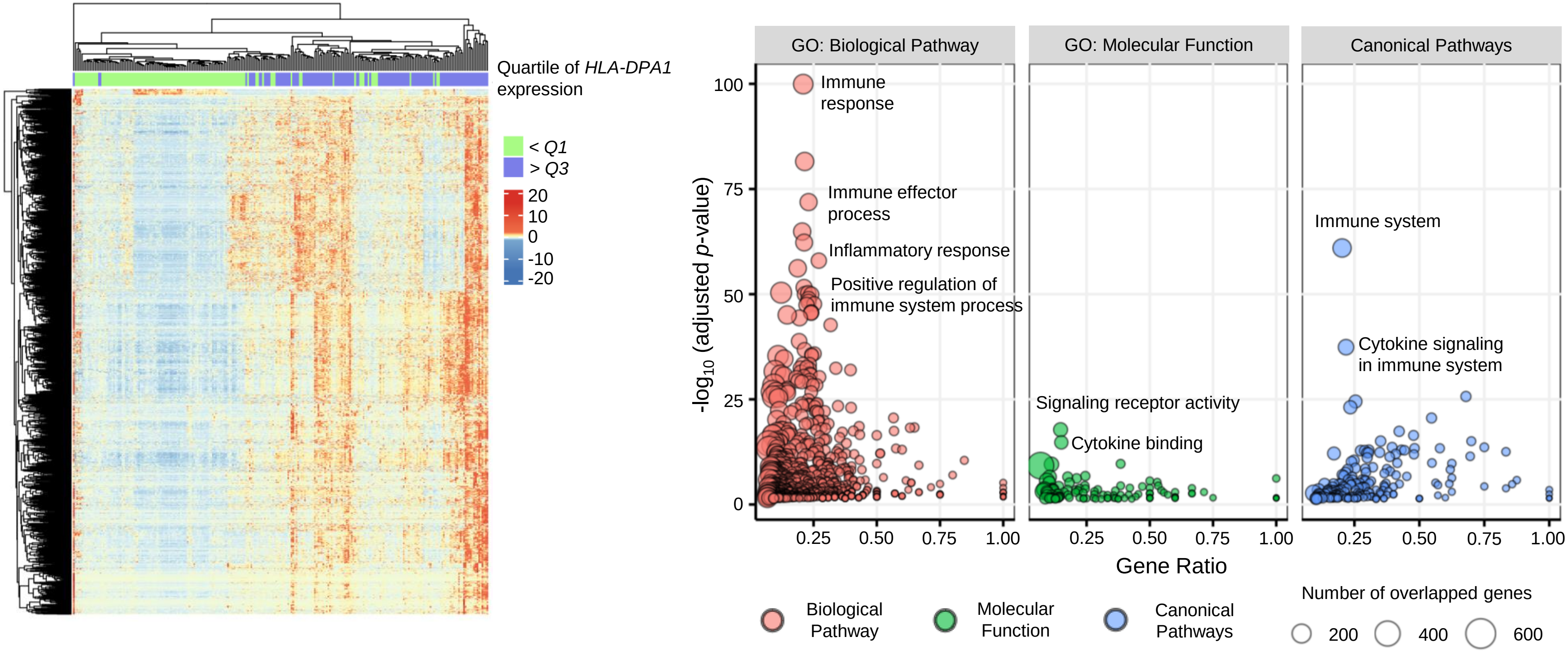

Figure S3. Genes whose expression is associated with *HLA-DPA1* transcript expression. (A) Expression heatmap of all detected transcripts. Q: quartile. (B) Gene Ontology terms and functional pathways enriched among associated genes.

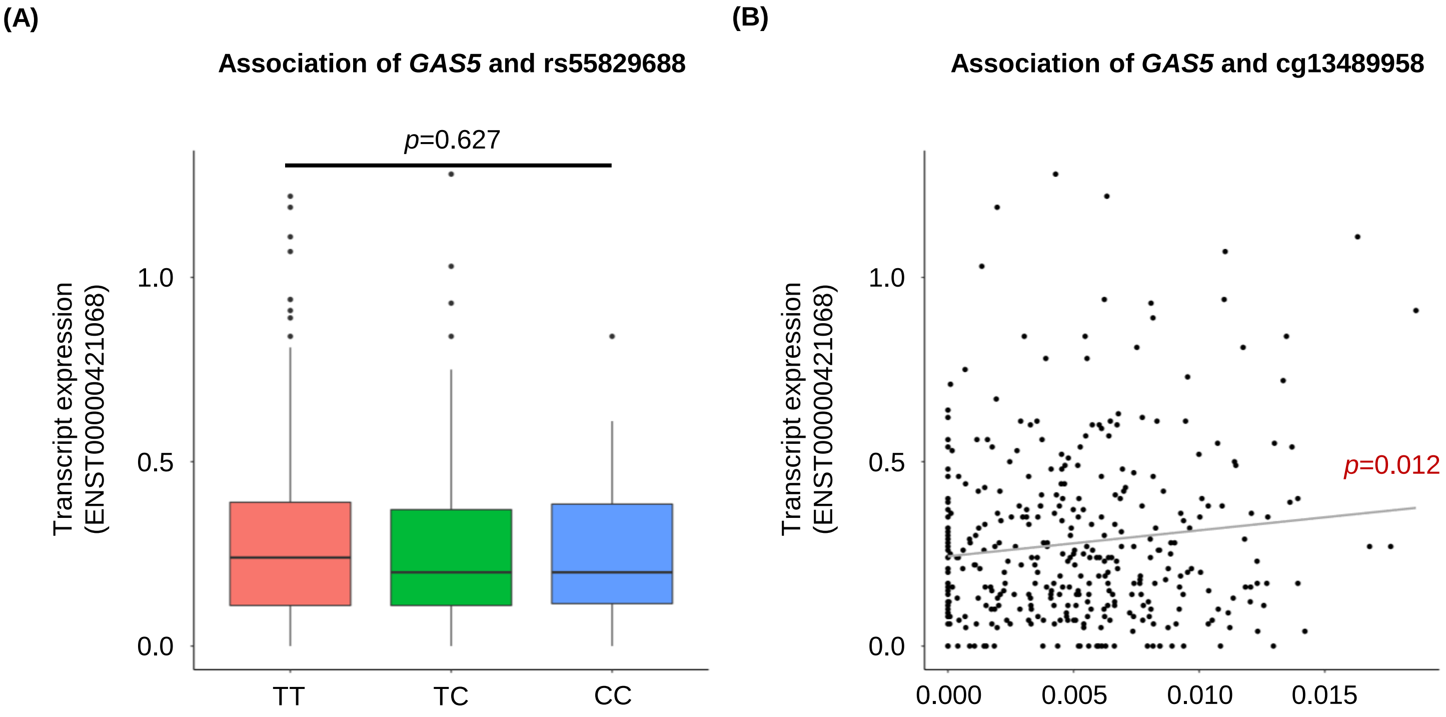

Figure S4. Association of *GAS5* transcript ENST00000421068 expression with either of (A) genotype at the SNP rs55829688 or (B) methylation status at the probe cg13489958.

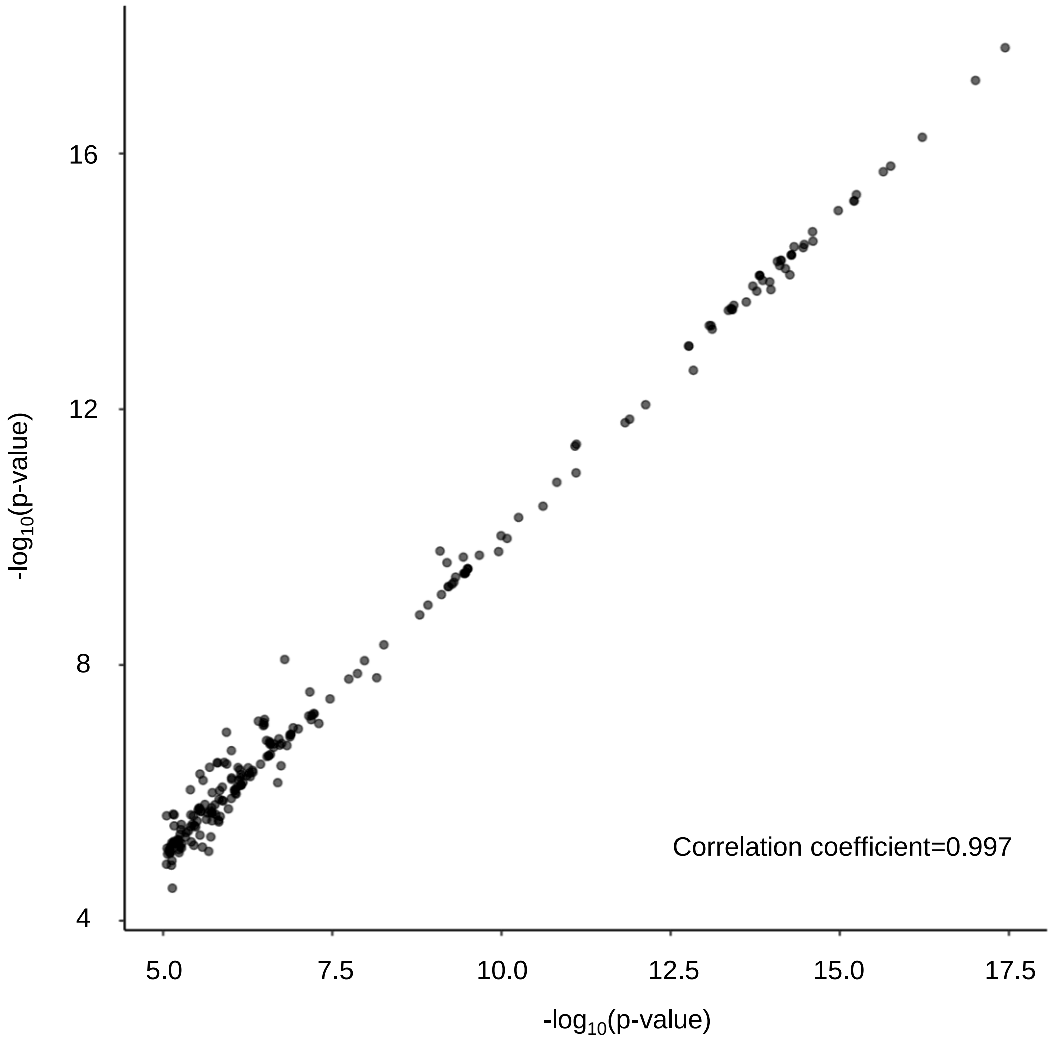

Figure S5. A scatter plot between the -$\log_{10}$(p-values) from the model with (x-axis) and without (y-axis) age/sex as covariates

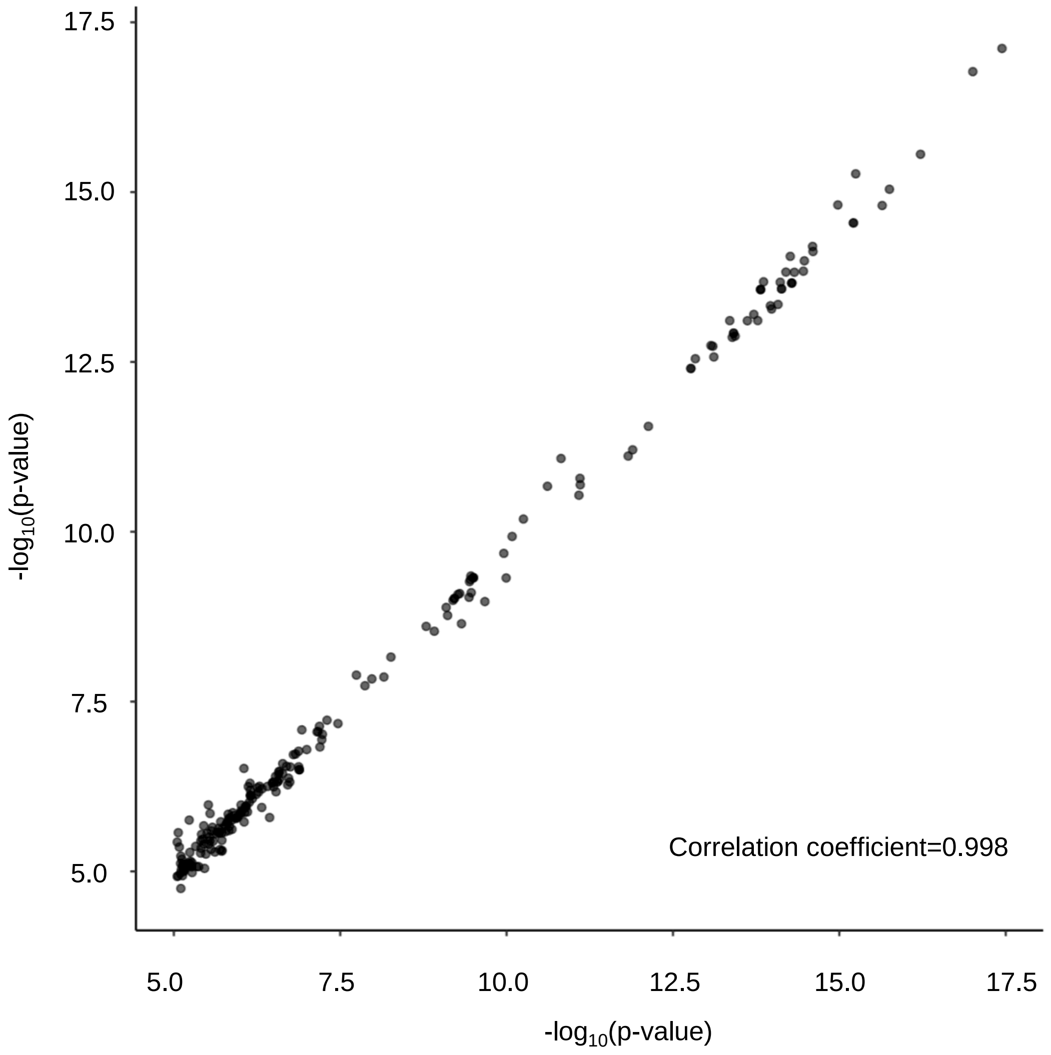

Figure S6. A scatter plot between the -$\log_{10}$(p-values) from the model with and without Braak stage, CEREAD, and APOE4 status as covariates

***IL1RAP* - rs73058141 and cg24004745**

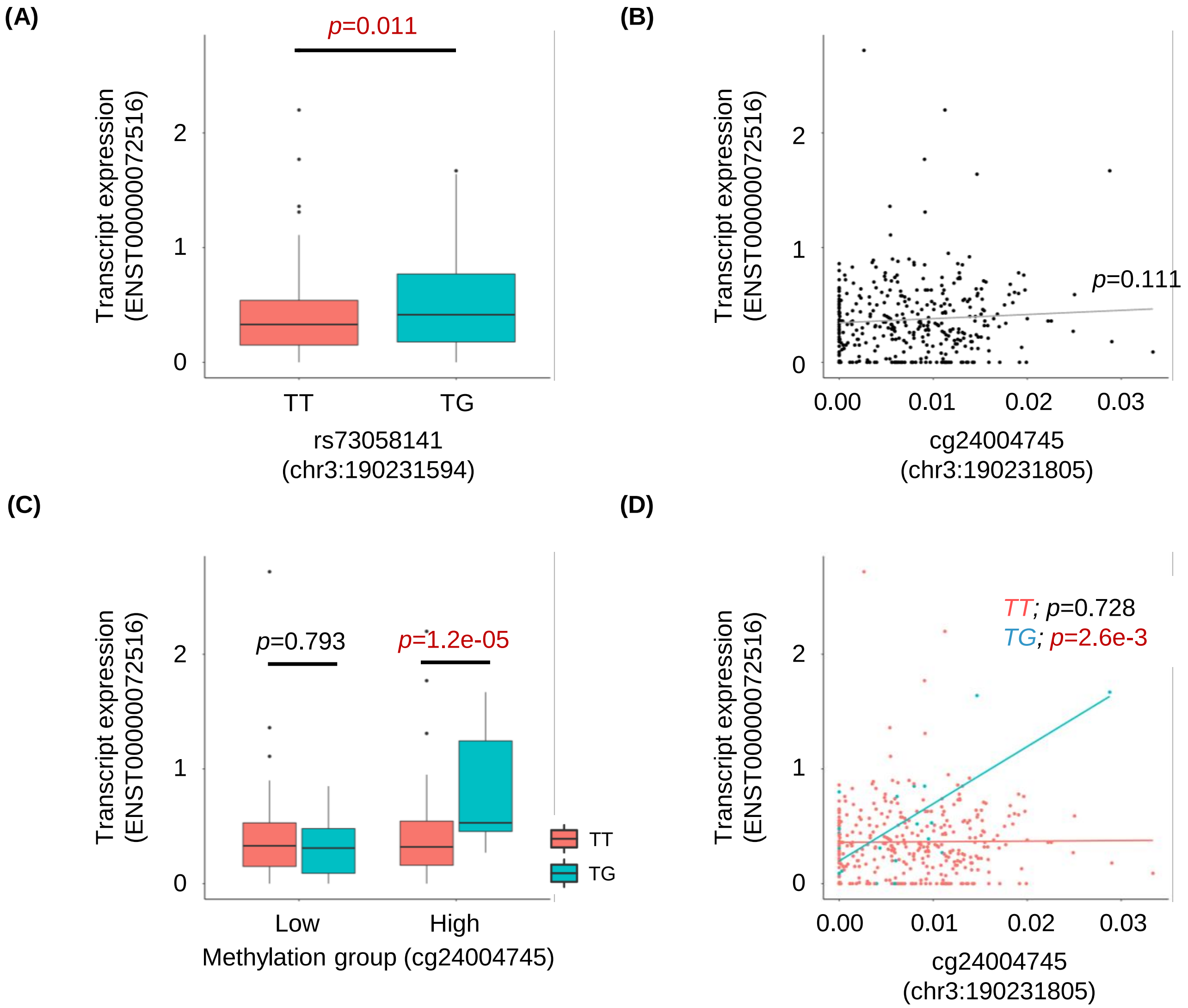

Figure S7. Interaction of the SNP rs73058141 and DNA methylation at cg24004745 on *IL1RAP* transcript expression (ENST00000072516). (A) Boxplot of transcript expression according to rs73058141 genotype. (B) Scatter plot of transcript expression association with methylation of cg24004745. (C) Boxplot of transcript expression distribution according to genotype among low- and high-methylation groups. K-means clustering was used to classify groups. (D) Scatter plot of transcript expression association with methylation level for each genotype.
